# Development of a Highly Sensitive and Specific Method for Detection of Circulating Tumor Cells Harboring Somatic Mutations in Non-Small-Cell Lung Cancer Patients

**DOI:** 10.1371/journal.pone.0085350

**Published:** 2014-01-21

**Authors:** Frank Breitenbuecher, Sandra Hoffarth, Karl Worm, Diana Cortes-Incio, Thomas C. Gauler, Jens Köhler, Thomas Herold, Kurt Werner Schmid, Lutz Freitag, Stefan Kasper, Martin Schuler

**Affiliations:** 1 Department of Medical Oncology, West German Cancer Center, University Hospital Essen, University Duisburg-Essen, Essen, Germany; 2 Institute of Pathology, West German Cancer Center, University Hospital Essen, University Duisburg-Essen, Essen, Germany; 3 Division of Thoracic Oncology, Ruhrlandklinik, West German Lung Center, University Duisburg-Essen, Essen, Germany; 4 Division of Interventional Pneumology, Ruhrlandklinik, West German Lung Center, University Duisburg-Essen, Essen, Germany; 5 German Cancer Research Center, Heidelberg, Germany; 6 German Cancer Consortium (DKTK), Heidelberg, Germany; University of Navarra, Spain

## Abstract

**Background:**

Oncogenic mutations are powerful predictive biomarkers for molecularly targeted cancer therapies. For mutation detection patients have to undergo invasive tumor biopsies. Alternatively, archival samples are used which may no longer reflect the actual tumor status. Circulating tumor cells (CTC) could serve as an alternative platform to detect somatic mutations in cancer patients. We sought to develop a sensitive and specific assay to detect mutations in the *EGFR* gene in CTC from lung cancer patients.

**Methods:**

We developed a novel assay based on real-time polymerase chain reaction (PCR) and melting curve analysis to detect activating *EGFR* mutations in blood cell fractions enriched in CTC. Non-small-cell lung cancer (NSCLC) was chosen as disease model with reportedly very low CTC counts. The assay was prospectively validated in samples from patients with *EGFR*-mutant and *EGFR*-wild type NSCLC treated within a randomized clinical trial. Sequential analyses were conducted to monitor CTC signals during therapy and correlate mutation detection in CTC with treatment outcome.

**Results:**

Assay sensitivity was optimized to enable detection of a single *EGFR*-mutant CTC/mL peripheral blood. CTC were detected in pretreatment blood samples from all 8 *EGFR*-mutant lung cancer patients studied. Loss of *EGFR*-mutant CTC signals correlated with treatment response, and its reoccurrence preceded relapse.

**Conclusions:**

Despite low abundance of CTC in NSCLC oncogenic mutations can be reproducibly detected by applying an unbiased CTC enrichment strategy and highly sensitive PCR and melting curve analysis. This strategy may enable non-invasive, specific biomarker diagnostics and monitoring in patients undergoing targeted cancer therapies.

## Introduction

Activating *EGFR* mutations define a group of genetically dependent pulmonary adenocarcinomas, which are exquisitely sensitive to EGFR tyrosine kinase inhibitors (EGFR-TKI). Therapeutic decisions in patients with metastatic NSCLC are guided by the *EGFR* mutation status, which is determined in tumor biopsies [1]. Acquisition of such biopsies may be hazardous to the patient. Moreover, a single tumor biopsy may not fully reflect the status of a metastatic cancer. Non-invasive methods for repetitive determination of prognostic and predictive genetic biomarkers could facilitate personalized cancer therapy.

Circulating tumor cells (CTC) have been described in several cancer entities. Enumeration of CTC has been correlated with clinical outcomes and treatment response [2]–[6]. These studies have applied immunocytochemical detection of protein markers, mostly neglecting genomic biomarkers such as *EGFR* mutation status. In contrast to leukemias, where malignant cells are abundantly present in the peripheral blood, CTC are rare in patients with solid tumors and a large variability of CTC counts has been observed [3], [5], [7]–[9]. CTC detection based on epithelial markers such as EpCAM or cytokeratins (CK) may overlook tumor cells undergoing epithelial-mesenchymal transition (EMT) [10] .

Here we describe a novel highly sensitive and specific strategy to detect CTC harboring somatic mutations in NSCLC patients. As a proof-of-concept model we have used in-frame deletions in the *EGFR* exon 19 (DelEx19), which comprise approximately 48% of all *EGFR* mutations [11]. We were able to detect *EGFR* DelEx19-mutated CTC prior to therapy in all patients with *EGFR* mutational status known from tumor biopsies that were assessed. Moreover, clearance of mutation-positive CTC correlated with treatment response and disease control.

## Materials and Methods

### Genomic DNA preparation

Genomic DNA was isolated from PBMNC and cell lines using the NucleoSpin® Blood Kit (Macherey-Nagel, Düren, Germany). If necessary, genomic DNA was amplified using the REPLI-g Midi Kit (QIAgen, Hilden, Germany). Genomic DNA from cell lines or plasmid DNA (pcDNA3.1V5/HisTOPO, Clontech, Mountain View, USA) encoding a human *EGFR* Exon 19 cDNA sequence harboring a 15 bp deletion (delE746-A750) were serially diluted. Cell lines A431 (*EGFR* wild type, wt) and NCI-HCC-827 (*EGFR* delE746-A750) were obtained from DSMZ (Braunschweig, Germany) and *EGFR* mutation status was verified by Sanger sequencing.

### PCR amplification and *EGFR*DelEx19 mutation detection

*EGFR* DelEx19 mutations were detected by melting curve analysis on a LightCycler 480 (Roche, Mannheim, Germany). Optimal mutation detection sensitivity was achieved by a combination of specifically designed hybridization probes imperfectly binding to EGFR Exon 19 sequences harboring a deletion at amino acid position 746 or 747, locked-nucleic acids (LNA) suppressing amplification of wildtypic *EGFR* sequences and preventing hybridization of probes to wildtypic *EGFR* Exon 19 sequences and finally applying asymmetric PCR conditions preferentially amplifying the DNA strand hybridization probes bind to. All parameters were empirically optimized to achieve optimal assay sensitivity. Each reaction (20 µl) contained 50 ng genomic DNA, 2 pmol forward and 2 pmol reverse primer (Eurofins MWG, Ebersberg, Germany; Ex19S: 5′-GTCTTCCTTCTCTCTCTGTCATAGGG-3′, Ex19R: 5′-GGGCCTGAGGTTCAGAGC-3′), 2 µl LightCyclerFastStart DNA Master HybProbe (Roche, Mannheim, Germany), 3 pmol hybridization probe 1 (“anchor”: 5′- LC640-ATTTTAACTTTCTCACCTTCTGGGATCCAG-PH) and 3 pmol hybridization probe 2 (“sensor”: 5′- TAATTCCTTGATAGCGACGGG-FL) (TIBMolbiol, Berlin, Germany). All PCR reactions were carried out with and without addition of 6 pmol locked-nucleic acid (LNA: 5′-TAATTCCTTGATAG-NH2; TIBMolbiol, Berlin, Germany).

### Immunomagnetic enrichment of circulating tumor cells from peripheral blood

Peripheral blood (20 mL) was collected in sodium citrate tubes (Sarstedt, Nümbrecht, Germany). PBMNC were isolated by density gradient centrifugation. The median enrichment of WBC by density gradient centrifugation was approximately threefold. EpCAM-positive cells and CD45-negative cells were enriched in two parallel reactions using anti-EpCAM (CD326) and anti-CD45 microbeads (Miltenyi Biotech, Bergisch Gladbach, Germany). Genomic DNA was isolated from the resulting four sample fractions (CD45^+^, CD45^−^, CD326^+^ and CD326^−^) and subjected to real time-PCR and melting curve analysis.

### Mutation detection by sequence analysis

For sequence analysis, genomic DNA of selected samples was amplified by whole genome amplification (WGA) using the REPLI-g Midi Kit (QIAgen, Hilden, Germany) according to the manufacturer's instructions. All sequence reads were generated by a commercial sequencing service (LGC Genomics, Berlin, Germany) on a Roche 454 GS FLX+ Titanium (Roche, Branford, USA) and analyzed using CLC genomics workbench (CLCbio, Aarhus, Denmark). Reads were imported into CLC genomics workbench (using *.fna and *.qual data), trimmed and mapped to a reference including *EGFR* exon 19 sequence as well as 50 bp up- and downstream intron sequences. The median coverage for exon 19 sequences was 7,316 reads (range 3,717–17,368).

### Patient samples/Ethics statement

Peripheral blood samples were obtained from patients with *EGFR* mutant and *EGFR* wt NSCLC following written informed consent. The study was approved by the Ethics Committee of the Medical Faculty of the University Duisburg-Essen (AZ. 10-4359).

### Statistics

Exploratory statistical analyses were conducted using GraphPad Prism 4 (GraphPad Software, La Jolla, USA) and IBM SPSS Statistics version 19 (IBM, Armonk, USA).

## Results

### Sensitivity and specificity of mutation detection

**Figure 1:**
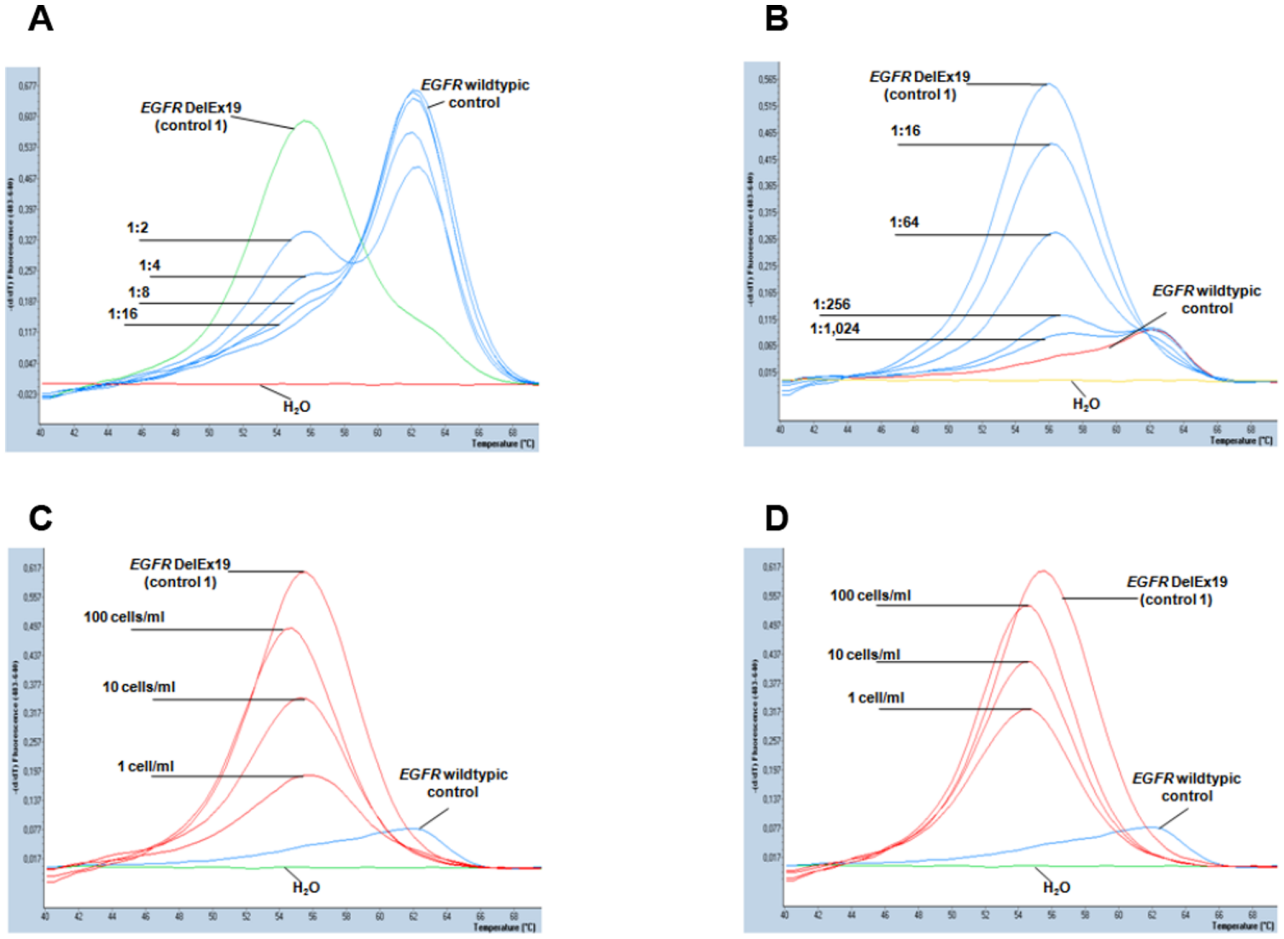
Sensitivity of *EGFR* DelEx19 mutation detection by real-time polymerase chain reaction and melting curve analysis. A) *EGFR* DelEx19 mutation detection in serially diluted DNA (50 ng/reaction) from A431 cells (*EGFR*-wild type control) and NCI-HCC-827 cells (*EGFR* DelEx19 mutant control 1). Melting peaks indicative of *EGFR*-wild type DNA (right) and *EGFR* DelEx19 (left) can be clearly distinguished. Real-time PCR reactions were carried out without addition of locked nucleic acids (LNA) and in serial DNA dilutions of up to 1∶16. Water (H_2_O, bottom line) and and 50 ng of undiluted genomic DNA *EGFR*-wild type (A431) and *EGFR*-mutant cells (NCI-HCC-827) were included as controls (representative examples of duplicate reactions). B) Reactions were conducted as in (A) but with addition of LNA (6 pmol). Note suppression of the *EGFR*-wild type signal, which allowed discrimination of the *EGFR* DelEx19 mutation signal up to a dilution of 1∶1,024 (<0.1%). C, D) Normal peripheral blood samples were spiked with defined numbers (0, 1, 10, 100 cells/mL) of *EGFR* DelEx19 mutant NCI-HCC-827 cells. Following immunomagnetic enrichment genomic DNA was extracted and subjected to real-time PCR and melting curve analysis including controls as in (B). Representative results of genomic DNA isolated from leucocyte-depleted cell fractions (CD45^−^, C) or CD326-enriched cell fractions (CD326^+^, D) are shown. In both cell fractions *EGFR* DelEx19 mutation signals can be clearly distinguished from the *EGFR*-wild type background up to a sensitivity threshold of 1 cell/mL. No mutation signals were detected in CD45^+^ or CD326^−^ cell fractions (data not shown).

In order to determine the threshold for *EGFR* DelEx19 mutation detection by melting curve analysis, we initially studied serial dilutions of genomic DNA from *EGFR* wt A431 cells and NCI-HCC-827 cells harboring an *EGFR* DelEx19 mutation (Figure 1a). By optimization of PCR parameters and inclusion of wt *EGFR*-specific LNA the sensitivity of detection was enhanced to 0.1% *EGFR*DelEx19-mutant sequences (Figure 1b). In further serial dilutions of plasmid DNA (pcDNA3.1.EGFRdelE746-A750) the mutation detection threshold was as low as 0.01% (data not shown).

Next, we spiked blood samples from healthy volunteers with defined cell numbers of NCI-HCC-827 cells. Samples were processed as described to enrich spiked tumor cells and all resulting cell fractions were subjected to genomic DNA isolation, real time PCR and melting curve analysis. In these experiments the reproducible lower limit of detection was 1 *EGFR*-mutant cell per 1 mL peripheral blood (Figure 1 c, d). This protocol was then applied to blood samples from three NSCLC patients with *EGFR* wildtypic tumors and two healthy volunteers. DNA isolated from all cell fractions tested negative for *EGFR* DelEx19 mutations (Suppl. Figure S1 a–e).

### Assay validation in clinical samples

To validate our strategy for CTC-enrichment and mutation detection we prospectively obtained peripheral blood samples from 8 patients with metastatic NSCLC harboring *EGFR* DelEx19 mutations, treated at our center within the LUX-Lung 3 study (EudraCT-No. 2008-005615-18; [12]). Per study inclusion criteria all patients had histologically confirmed, medically untreated stage IIIB/IV pulmonary adenocarcinoma (Table 1). All *EGFR* mutations were confirmed by central analysis.

**Table 1:** Patient data and demographics.

Pat. ID	Sex	Age (years)	*EGFR*mut. tumor biopsy	*EGFR*mut. CTC	CTC detection baseline	CTC clearance	Days on study medication (TTF)	Best treatment response	Study medication
018	m	50	DelEx19	DelEx19	Y	N	84	SD	P/C
015	f	61	DelEx19	DelEx19	Y	Y	252	PR	A
042	f	66	DelEx19	DelEx19	Y	N	148	SD	P/C
021	f	67	DelEx19, T790M	DelEx19	Y	Y	189	SD	A
041	m	65	DelEx19	DelEx19	Y	Y	436	PR	A
017	m	53	DelEx19	DelEx19	Y	Y	1,086	PR	A
016	f	42	DelEX19/L858R*	DelEx19	Y	N	173	PD	A
030	f	64	DelEx19	DelEx19	Y	N	84	PR	P/C

Demographics, treatment and outcome of the validation cohort comprising patients with stage IV pulmonary adenocarcinoma. All mutation analyses on tumor samples were conducted by a central laboratory within the LUX-Lung 3 study. Abbreviations: m, male; f, female; TTF, time to treatment failure; SD, stable disease; PR, partial response; PD, progressive disease; P/C, Pemetrexed/Cisplatin; A, Afatinib.

EGFR mutations detected by central laboratory analysis and additional local mutation testing.

A blood sample was designated “negative” for *EGFR*-mutant CTC if no mutation signal was obtained by real time PCR analysis of genomic DNA isolated from all cell fractions (CD45^+^, CD45^−^, CD326^+^ and CD326^−^) obtained at the respective time point. Based on our titration experiments this indicated that *EGFR*-mutant CTC dropped below 1 cell/mL blood. In case an *EGFR* DelEx19 mutation was detected in genomic DNA from at least one cell fraction the sample was declared “positive” for *EGFR*-mutant CTC.

**Figure 2:**
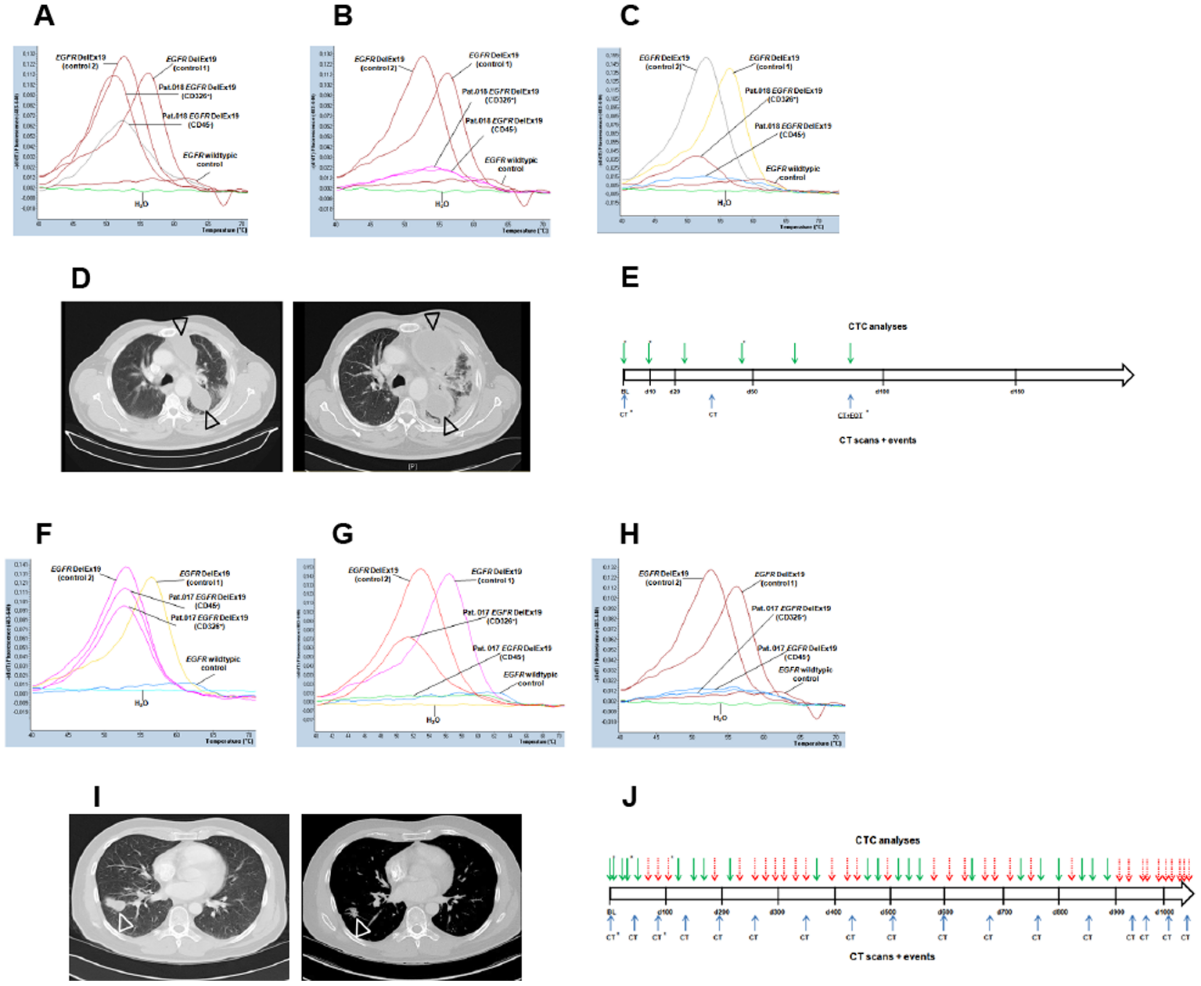
Detection of *EGFR*-mutant CTC and treatment outcome in patients with NSCLC harboring *EGFR* DelEx19 mutation. Representative examples of *EGFR*-mutant CTC analyses and clinical course of patients of the prospective validation cohort. All patients were treated within the LUX-Lung 3 study for stage IV *EGFR*-mutant NSCLC. Treatment course, imaging time points (CT) and time points of CTC analyses are illustrated in **E** and **J**. Solid arrows indicate “CTC positivity”, dashed arrows “CTC negativity”. Asterisks indicate time points of the CTC analyses/CT scans shown in **A–D**, and **F–I**. (**A**) Base line analysis of patient 018 showed strong *EGFR* DelEx19 signals in two cell fractions (CD326^+^and CD45^−^). (**B**, **C**) Sequential analyses in patient 018 reveal strong *EGFR* DelEx19 signals at least in one relevant cell fraction. (**D**) Representative CT images from patient 018 at base line, and at day 84 of study treatment confirming progressive disease. (**F**) Base line analysis of patient 017 showing strong *EGFR* DelEx19 signals in two cell fractions (CD326^+^and CD45^−^). (**G**, **H**) Sequential analysis in patient 017 showing decreased (**G**) and negative (**H**) *EGFR* DelEx19 signals in both relevant cell fractions. (**I**) Representative CT images from patient 017 at base line, and at day 84 of study treatment confirming a partial response.

Base line samples were obtained within 7 days prior to study therapy, sequential blood samples were taken at regular intervals under treatment and follow-up. Upon clinical progression and/or end of therapy an additional blood sample was obtained. In total 148 blood samples were collected over a period of 36 months and analyzed for the presence of *EGFR*-mutant CTC (median: 12 samples/patient; range 7–56). Base line blood samples from 8 of 8 patients (100%) were “positive” for *EGFR*-mutant CTC. Interestingly, sequential samples from 4 of 8 patients turned “negative” for *EGFR*-mutant CTC within a median of 34 days (range: 20–84 days) of study treatment (Figure 2 a–j). “CTC negativity” (i.e. less than 1 *EGFR* DelEx19-mutated cell per milliliter blood) persisted for a median of 109 days (range: 35–199 days). Genomic DNA from samples displaying a strong *EGFR* DelEx19 mutation signal in our newly developed assay (shown in Figures 2A–2C and 2F), was analyzed on a Roche 454 GS FLX+ Titanium sequencer. To our surprise, in none of these samples a deletion in *EGFR* Exon 19 could be detected (data not shown).

### Association of EGFR mutant CTC with clinical outcomes

**Figure 3:**
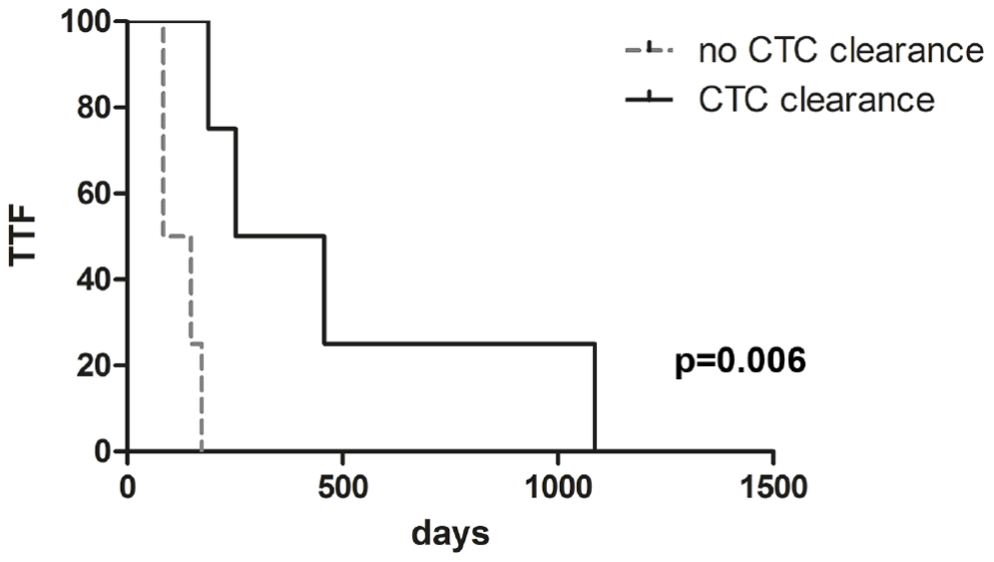
Clearance of *EGFR*-mutant CTC correlates with time to treatment failure. Kaplan-Meier analysis comparing TTF of patient groups with and without clearance of CTC during therapy. Patients were grouped according to “clearance” (median TTF 355 days) or “non-clearance” (median TTF 116 days) of *EGFR*-mutant CTC under treatment with afatinib or pemetrexed/cisplatin. Groups were compared by explorative Kaplan-Meier analysis (p = 0.006, Log-rank test).

Based on this observation we queried the association of *EGFR*-mutant CTC with treatment response and duration in our validation cohort. We defined a patient as “*EGFR*-mutant CTC cleared” if two consecutive blood samples were tested “negative”, i.e. *EGFR*-mutant CTC counts dropped below 1 cell/mL. “CTC recurrence” was declared when two consecutive blood samples tested “positive” for *EGFR*-mutant CTC. Results from CTC mutation analysis were correlated with radiological response assessed within the LUX-Lung 3 study. All 4 patients who “cleared” *EGFR*-mutant CTC achieved disease control, with 3 partial responses (PR) and one stable disease (SD) per RECIST. In 4 patients with persisting CTC in their peripheral blood and who thus failed to “clear” *EGFR*-mutant CTC only one objective response (PR) was observed, while 2 patients achieved SD and one patient progressive disease (PD). At follow-up all 4 patients with “clearance” of *EGFR*-mutant CTC developed PD. *EGFR*-mutant CTC returned to “positive” at a median of 140 days (range 0–960 days) prior to radiologically documented PD. Hence, an increase in *EGFR*-mutant CTC counts above the threshold of 1 cell/mL could be an early indicator of disease relapse and resistance in patients that have “cleared” CTC. By exploratory statistical analyses we observed a significant association (Spearman-Rho correlation coefficient 0.868, p<0.01) between the duration of “CTC negativity” and the time to treatment failure. Patients who had “cleared” *EGFR*-mutant CTC below the level of detection showed a significantly prolonged time to treatment failure (median: 355 days; range: 189–1,086 days; p = 0.006, Log-rank test) as compared to patients who remained “positive” for *EGFR*-mutant CTC (median: 116 days, range: 84–173 days; Fig. 3).

## Discussion

Currently, the detection of genetic aberrations in tumor tissues is the clinically most powerful biomarker for stratification of “targeted” pharmacotherapies in metastatic lung cancer [13], [14]. Treatment with gefitinib and crizotinib, as well as first-line erlotinib, is conditional on the demonstration of *EGFR* mutations and *ALK* rearrangements. Mutation detection is frequently performed in small and/or archival tumor specimens, which may not reflect the current genomic profile at treatment initiation [15]. Moreover, the mutational spectrum of a cancer changes under the selective pressure of a given therapy, which can only be revealed by sequential biopsies. Against this background, the detection of cancer-specific mutations in other formats than tumor biopsies has gained interest. In NSCLC, successful detection of mutated DNA sequences was reported in serum, plasma or bronchioalveolar lavage fluid [16]–[19]. Nevertheless, most of these reports displayed a level of sensitivity below clinical requirements.

The early systemic dissemination of cancer cells is considered of biological relevance. Using primary antibodies against epithelial markers, disseminated tumor cells and CTC have been detected in bone marrow and blood from patients with various malignancies. Among a large variety of alternative strategies applied for CTC-enrichment and analysis, an automated system for immunocytometric detection and quantitation of CTC has been developed, which was validated in breast, prostate, colorectal and lung cancer patients [2], [5], [8], [20]–[23]. Interestingly, the absolute amount of CTC detected in advanced stage cancer patients varied considerably, depending on CTC-isolation techniques. While high numbers of CTC were detected in patients with small-cell lung cancer, absolute CTC numbers and dynamic range in NSCLC seemed too low for broad clinical applicability. Nevertheless, it has become increasingly clear that CTC populations are phenotypically heterogenous and epithelial-marker independent enrichment approaches are warranted to display the full picture of CTC in a cancer patient at a given time point. Several recent reports supported this notion by consistently showing higher CTC numbers when comparing epithelial-marker-independent CTC enrichment techniques (e.g. enrichment by cell size) with the widely applied CTC isolation by selection of EpCAM and CK-positive cells using the CellSearch system [5], [8], [24], [25].

Against this background we reasoned whether probing for cancer-specific somatic mutations instead of microscopy-based parameters enhances the sensitivity of CTC detection in NSCLC. We have chosen *EGFR* DelEx19 mutations as a model to develop a highly sensitive, specific and robust method for mutation detection in mixtures of genomic DNA that were extracted from CTC-enriched peripheral blood cell populations. The novel method applied in this study has been designed to detect the vast majority of *EGFR* DelEx19 mutations, notably all possible mutant variants disrupting codons 746 and 747 of the *EGFR* gene. In contrast to a prior study analyzing only EpCAM-positive blood cells [26] we have chosen an unbiased, epithelial marker-independent CTC enrichment strategy where samples were split and CTC enrichment was achieved by a dual approach, depleting leucocytes in one half of a sample and selection of EpCAM-positive cells in the other half. This takes into account the phenotypic variability of CTC, which may lose one or several epithelial markers while being shed from a primary tumor or metastases [27]–[29]. In pilot experiments, Immunofluorescence staining of selected CTC samples from NSCLC patients indeed showed heterogeneous populations of CTCs displaying epithelial, mesenchymal and mixed phenotypes (data not shown). Applying this unbiased CTC-enrichment approach, we achieved a 100% detection rate in a pilot cohort of 8 patients with *EGFR*-mutant NSCLC. Most interestingly, we observed an association of *EGFR*-mutant CTC with treatment response and outcome. These findings are most likely to be attributed to the novel and highly sensitive mutation detection method applied in this study. It has been recognized for some time that detection of mutations at levels below 1% is not reliably possible by next generation sequencing applications due to intrinsic error rates of this technology [30]. Only very recently improved technologies were reported, rendering detection of mutations at levels of 0,1% and below possible [31], [32]. In keeping with these results, we were not able to detect *EGFR* Del19 mutations by next generation sequencing in genomic DNA of samples displaying unambiguous mutation signals in our newly developed mutation detection assay. These findings indicate that our newly developed mutation detection assay apparently displays higher sensitivity than standard next generation sequencing techniques. Another remarkable aspect of our mutation detection assay was the observation that different *EGFR* DelEx19 mutations gave rise to different melting curves (see Figure 2 and suppl. Figure S1). This is most likely due to the characteristics of one of the hybridization probes, which is designed to bind to *EGFR* Exon 19 wildtypic sequences in the region of potential deletions leading to the dissociation of the probe during melting curve analysis at different temperatures depending on the sequence of the respective deletion.

While these results are promising, we are aware that the patient population analyzed here is very small and validation in larger patient populations including additional mutations is required. Currently, equally sensitive mutation detection assays covering multiple therapy-relevant regions of the *EGFR* gene (e.g. codons L858 and T790) and other oncogenes are being developed.

In summary, we have described a novel strategy based on CTC enrichment and highly sensitive detection of somatic mutations, which can be applied for mutational profiling and monitoring of treatment efficacy in patients with *EGFR*-mutant NSCLC. By assay optimization we have shown that the problem of notoriously low CTC counts in stage IV NSCLC can be overcome. This may be another step toward the vision of a “liquid biopsy” for hazard-free molecular diagnostics and disease monitoring in patients with metastatic lung cancer.

## Supporting Information

Figure S1 ***EGFR*** **DelEx19 mutation analysis in blood samples from NSCLC patients with** ***EGFR*** **–wild type tumors and healthy control persons.** Peripheral blood samples from three patients with*EGFR*-wild type NSCLC (A, B, C) and two healthy volunteers (D, E) were enriched, separated in cellular fractions and subjected to DNA isolation as described. Mutation detected was conducted by real-time PCR and melting curve analysis in the presence of LNA. No *EGFR* DelEx19 signal was detected. H_2_O (bottom line) and 50 ng of undiluted genomic DNA of NCI-HCC-827 cells (“*control 1*”), plasmid DNA containing an *EGFR* DelEx19 sequence (“control 2”) and A431 cells (“*EGFR wildtypic control*”) were included as negative and positive controls. For clarity only single values of duplicates are shown.
(TIF)
