# Supplementary figures and images for: Development of a Highly Sensitive and Specific Method for Detection of Circulating Tumor Cells Harboring Somatic Mutations in Non-Small-Cell Lung Cancer Patients
Source: PLoS One. 2014 Jan 21;9(1):e85350. doi: 10.1371/journal.pone.0085350 (PMC3897440; doi:10.1371/journal.pone.0085350)

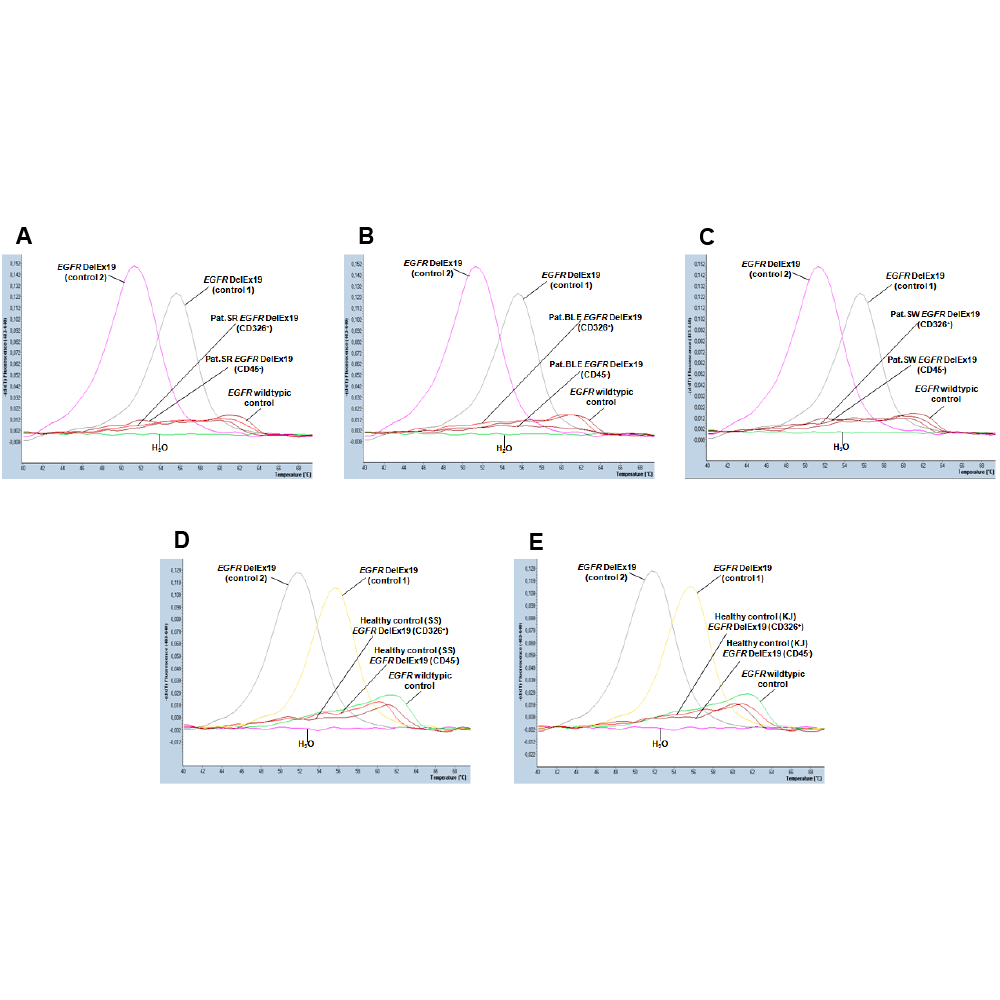

Supplement: Figure S1 — EGFR DelEx19 mutation analysis in blood samples from NSCLC patients with EGFR –wild type tumors and healthy control persons. Peripheral blood samples from three patients withEGFR-wild type NSCLC (A, B, C) and two healthy volunteers (D, E) were enriched, separated in cellular fractions and subjected to DNA isolation as described. Mutation detected was conducted by real-time PCR and melting curve analysis in the presence of LNA. No EGFR DelEx19 signal was detected. H2O (bottom line) and 50 ng of undiluted genomic DNA of NCI-HCC-827 cells (“control 1”), plasmid DNA containing an EGFR DelEx19 sequence (“control 2”) and A431 cells (“EGFR wildtypic control”) were included as negative and positive controls. For clarity only single values of duplicates are shown. (TIF) [file pone.0085350.s001.tif]
